# Shoot dominance relationships lead to robust reproductive outputs

**DOI:** 10.1093/plphys/kiab234

**Published:** 2021-07-29

**Authors:** Madeleine Seale

**Affiliations:** Department of Plant Sciences, University of Oxford, South Parks Road, Oxford, OX1 3RB, UK

Increasing fruit and seed yield is one of the most important aims for crop improvement and expansion of food production. Reproductive development arises from a series of developmental transitions of the shoot meristems, which may activate to produce inflorescences, flowers, and fruits, or arrest at any of these stages. (Hempel and Feldman, 1994; Walker and Bennett, 2019; Wang and Li, 2008). In this issue of *Plant Physiology*, Walker, Wheeldon, and Bennett have untangled the relationships between different aspects of reproductive architecture and concluded that both *Arabidopsis thaliana* and *Brassica napus* tend to maintain a consistent number of shoot inflorescences and fruits regardless of developmental trajectory.

In a scientific world of increasingly higher resolution, higher throughput, bigger data, and ever more complex molecular manipulations, it can be easy to forget that experimental elegance and sophistication can be still be achieved with technically straightforward methods. The authors altered resource availability by varying soil volume and used variations of classical decapitation experiments to understand how reproductive development is coordinated across the shoot system.

In both species examined, the inherent developmental characteristics, such as plant size and shoot architecture, determine reproductive allocation. These traits are also affected though by environmental factors, such as nutrients and resources (Wheeldon et al., 2021). With increased soil volume, there was an increase in the number of inflorescences and fruits produced. Limitations in reproductive allocation at low volume were not wholly due to nutrient availability though, as addition of fertilizers was only partially able to increase yields. This indicates that soil volume may act as a proxy for future nutrient availability, which plants use to cautiously judge investment in reproductive structures.

This finding highlights a complex problem for plants: they must decide how many inflorescences to produce, and thus determine reproductive capacity, weeks or months before flower and fruit production, despite potential differences in resource availability over time. The authors discovered that Arabidopsis and Brassica determine their inflorescence shoot number at an early stage but that this only weakly predicts the eventual number of fruits produced.

Remarkably, these early decisions persist and reproductive development is incredibly stable to perturbations of shoot architecture. Removing secondary inflorescences releases the inhibition of dormant meristems (Bangerth, 1989). This treatment generated almost the exact same number of inflorescence shoots by the time development arrested, regardless of the number or location of those that were originally excised. The resulting number of fruits was also similarly robust even when playing experimental whack-a-mole to continuously remove developing fruits. These data imply that, for a given set of resources, plants maintain a consistent number of reproductive organs via their famously iterative and plastic continuous development.

**Figure 1:**
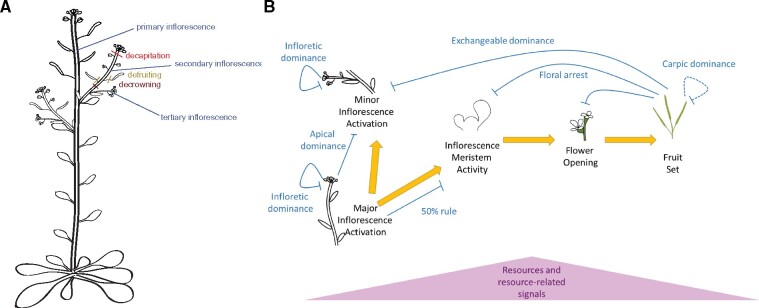
A, Schematic illustrating Arabidopsis shoot architecture and experimental manipulations. B, Schematic illustrating developmental trajectories and proposed dominance relationships between reproductive structures. Carpic dominance occurs in *B. napus* but not *A. thaliana*; resources and resource-related signals are thought to impact all stages of development. Modified from Walker et al. (2021).

Plasticity in shoot architecture is closely connected to apical dominance relationships between shoot meristems (Bangerth, 1989). To understand this further, Walker et al. (2021) carried out a set of architectural perturbations (Figure 1A). They found that decrowning (removing all reproductive organs), decapitating (removing inflorescence meristems and unopened flowers) and defruiting (removing fruits) all resulted in activation of additional inflorescence shoots. All reproductive organs appeared to additively contribute to inhibition of subsidiary reproductive meristems.

Interestingly, the dominance relationships appear to act locally within inflorescences (Figure 1B). Removal of reproductive organs led to increased tertiary or quaternary branches arising from individual secondary inflorescences but did not activate further secondary inflorescences. This indicates that as well as fractal-like patterns in organ form, the regulatory signaling mechanisms operate in a similarly iterative manner such that each inflorescence shoot system operates in an equivalent manner to the primary inflorescence. Tertiary and higher-order branches have typically been overlooked in studies of shoot branching, but this study highlights that they are as important in determining reproductive success as lower-order branches.

While most experimental conclusions were similar between Arabidopsis and Brassica, one key difference emerged for carpic dominance. This is the phenomenon by which fruits specifically inhibit the production of further fruits by reducing their size, or arresting their development at early stages (Walker and Bennett, 2018). This was clearly observed in Brassica but not in Arabidopsis despite the contribution of Arabidopsis fruits to overall dominance relationships within reproductive shoots.

The data presented provide a framework for understanding reproductive shoot architecture (Figure 1B) in Brassicaceae and open up a number of developmental and mechanistic questions. How and when is the reproductive organ number determined? How do plants detect soil volume? What are the signaling mechanisms underlying reproductive coordination? The latter seems likely to be integrated within the auxin–strigolactone–cytokinin signaling mechanisms already well-described for apical dominance (Barbier et al., 2019; Domagalska and Leyser, 2011; Waldie et al., 2014), but this has not been extensively investigated in terms of reproductive architecture.

The contribution of reproductive organs to shoot architecture dominance has often been assumed but rarely investigated. It appears that different organs communicate with precisely balanced signals to maintain a robust reproductive output despite often being located on distinct parts of the plant. This study highlights that there are quantitative differences in the inhibitory capacity of different reproductive elements and it will be interesting to find out whether this is due to organ size, auxin production, activity as a nutrient sink or source, or combinations of multiple signals. If these signals can be understood better, this may open the door to breaking down these tight dominance relationships and predetermined organ number, to ultimately improve yields.
